# Total gastrectomy in systemic scleroderma when anti-reflux surgery is not viable: A case report

**DOI:** 10.1016/j.ijscr.2019.08.022

**Published:** 2019-08-20

**Authors:** Carlos Jose Perez Rivera, Akram Kadamani Abiyomaa, Alejandro González-Orozco, Maria Antonia Ocampo, Isabella Caicedo, Manuel Santiago Mosquera

**Affiliations:** Fundación Cardioinfantil – Instituto de Cardiología, Bogotá, Colombia

**Keywords:** ISHLT, International Society of Heart and Lung Transplantation, GERD, gastroesophageal reflux disease, SCARE, Surgical Case Report, GERD-HRQL, Gastroesophageal Reflux Disease Health Related Quality of Life, Pulmonary transplantation, Scleroderma, Gastroesophageal reflux disease, Gastrectomy Roux-en-Y

## Abstract

**Introduction:**

Systemic scleroderma is an autoimmune disease that can affect the respiratory system and the gastrointestinal tract. When diffuse lung disease and pulmonary hypertension develop, a lung transplant is usually considered as treatment. This option, however, is not feasible in the presence of concomitant gastroesophageal reflux disease. In this case, medical therapy is initially warranted. If this fails, surgical approach may be considered in order for the patient to be a lung transplant candidate.

**Case presentation:**

A 56-year-old female, with previous history of intestinal pneumonitis, mild pulmonary hypertension and gastroesophageal reflux secondary to systemic scleroderma, is considered for lung transplant. Initially, due to persistent gastroesophageal reflux, a transplant was not a viable. This was corrected with an open gastrectomy with roux-en-Y anastomosis. Follow-up one week later revealed normal anatomy, adequate esophageal-jejunal anastomosis, and adequate contrast medium transit via esophagogram. Additionally, there was no evidence of contrast medium reflux indicating a resolved gastroesophageal reflux disease. This led to the patient becoming a candidate for lung transplant.

**Discussion:**

We suggest an open gastrectomy with roux-en-Y anastomosis as an alternative to the Nissen fundoplication for patients with connective tissue disease that develop terminal pulmonary consequences and require a lung transplant.

## Highlights

• Systemic scleroderma in severe cases, such as gastroesophageal reflux disease, a lung transplant cannot be performed.
• Although gastroesophageal reflux disease can be medically treated, a poor response will warrant an anti-reflux surgery.
• We propose an open gastrectomy with roux-en-Y anastomosis as an alternative to the Nissen fundoplication.
• The decision to provide a surgical intervention must be individualized.

## 1 Introduction

Scleroderma is a systemic autoimmune disease that affects the connective tissue and has life-threatening cardiovascular, gastrointestinal, and respiratory complications. Based on the 2006 International Society of Heart and Lung Transplant (ISHLT) guidelines, a lung transplant is warranted when the consequences produce diffuse interstitial lung disease and pulmonary hypertension. One of the contraindications of this is gastroesophageal reflux disease (GERD). This condition can be medically treated, but if this fails a surgical approach is often required to ensure eligibility for a lung transplant. The downside to the surgical approach is recurrence of disease, up to 21% in patients with connective tissue diseases [1]. We present the following case report as a successful alternative surgical therapeutic approach in these patients to ensure eligibility for a lung transplant. The following approach was completed in the Fundacion Cardioinfantil – Instituto de Cardiologia (IC), in Bogota, Colombia. This case report is written according to surgical case report (SCARE) criteria [2].

## 2 Case presentation

Patient is a 56-year-old female with body mass index of 22.5 kg/m^2^ and previous clinical history of diffuse scleroderma, intestinal pneumonitis, mild pulmonary hypertension, secondary gastroesophageal reflux, and severe esophageal motility disease. Receiving immunosuppression, antihypertensive, proton pump inhibitor and prokinetic management with weak response, patient is admitted for surgical management after a 1-year follow-up with cardiology, pneumology, and gastroenterology.

**Fig. 1:**
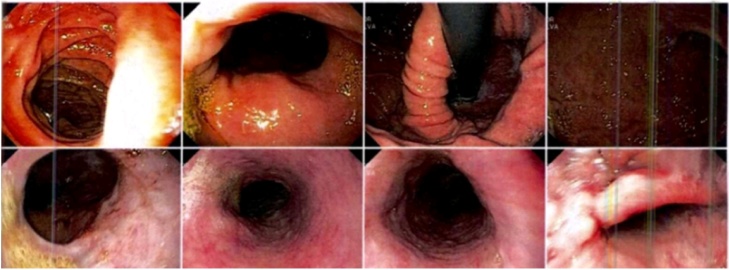
**Upper gastrointestinal endoscope image.** This esophageal image reveals and concludes an disfunction of the esophageal motility, esophagitis grade A (Los Angeles classfication), hiatal hernia, chronic corporal-antral gastritis, and mild bulbo-duodenitis.

At admission, patient was symptomatic with a previous diagnosis of esophageal aperistalsis (Fig. 1) and dysphagia with poor response to medical therapy. Physical examination revealed no significant findings. The lung transplant surgical team determined the patient was not a candidate given the severity of the GERD, due to a high risk of primary graft dysfunction. This led to a medical committee deciding an open total gastrectomy with roux-en-Y anastomosis prior to lung transplant for this particular case. This case report reveals a surgical alternative in patients with GERD secondary to scleroderma despite a high risk of disease recurrence.

**Fig. 2:**
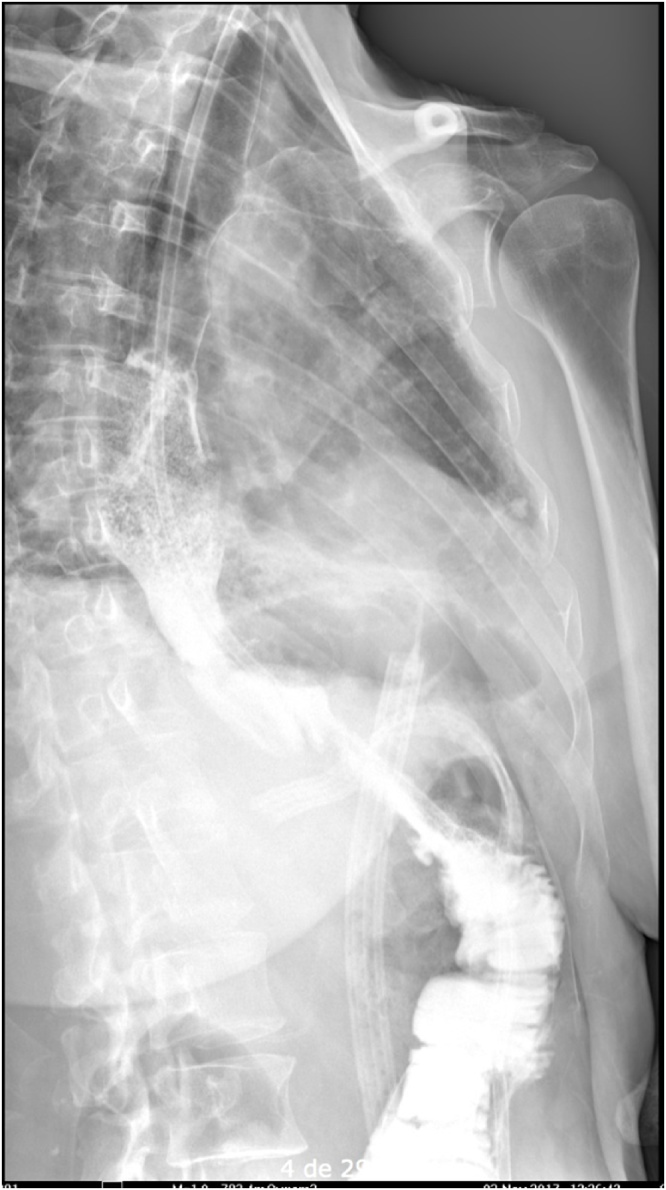
**Esophagogram image.** This esophageal image reveals normal morphology, without stenosis or motility difficulties, adequate anastomosis diameter, an adequate transit of the medium without extravasation or reflux.

**Fig. 3:**
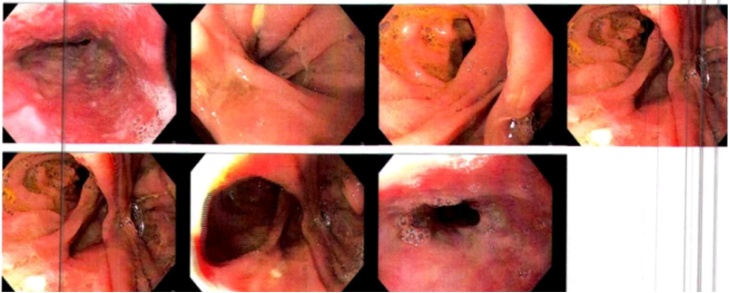
**Upper gastrointestinal endoscope image following balloon dilations.** This esophageal image reveals and concludes an adequate anastomosis, partial stenosis with posterior esophageal balloon dilation of 11 mm.

**Fig. 4:**
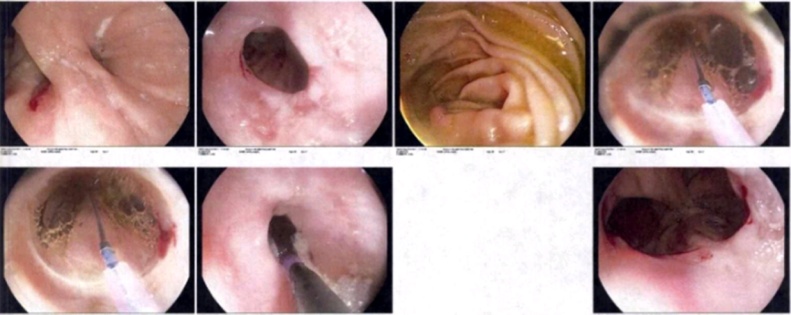
**Upper gastrointestinal endoscope image following balloon dilations.** This esophageal image reveals and concludes an adequate anastomosis with discrete stenosis with posterior esophageal balloon dilation of 15 mm.

**Fig. 5:**
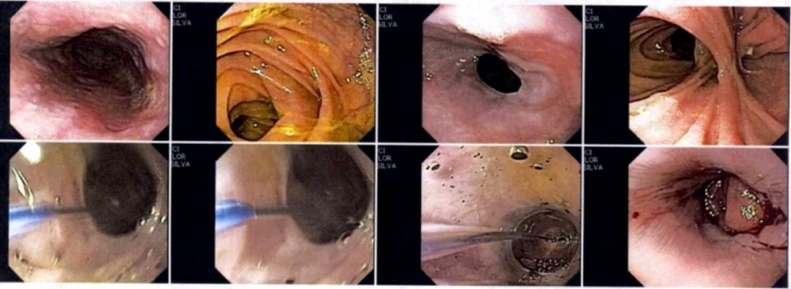
**Upper gastrointestinal endoscope image following balloon dilations.** This esophageal image reveals and concludes an adequate anastomosis with partial stenosis with posterior esophageal balloon dilation of 18 mm. Additionally, it reports multiple secondary mucosal tears self-limited and a Barrett's esophagus impression.

Follow-up one week later with esophagogram revealed normal esophageal morphology, no stenosis or motility difficulties, adequate esophago-jejunal anastomosis diameter, no extravasation of the contrast medium and an adequate transit of the medium to the small intestine. There is no evidence of reflux of the medium (Fig. 2). In out-patient consult, an upper gastrointestinal endoscopy is performed within a 3-month period which revealed mild esophago-jejunal anastomosis stricture resolving after three balloon dilations of 11 mm, 15 mm, and 18 mm, respectively (Fig. 3, Fig. 4, Fig. 5). Currently, the patient continues follow-up consults, with an adequate postoperative state, asymptomatic according to the Gastroesophageal Reflux Disease Health Related Quality of Life (GERD-HRQL) instrument and currently awaits lung transplant.

## 3 Discussion

Scleroderma is a connective tissue autoimmune disease which primarily affects the skin and other organs. The most commonly affected organs are the kidney, digestive tract, and the lungs. The major cause of mortality in scleroderma is a development of interstitial pulmonary disease [3]. In the digestive tract, the esophagus is affected in 50–80% of patients, with its primary effect problems in peristalsis and inferior esophageal sphincter damage [4]. In this case report, the patient had a terminal lung disease which required lung transplant. However, this patient was not a candidate due to concomitant GERD, given the high risk [5] between GERD and obliterating bronchiolitis syndrome leading to a primary graft dysfunction.

GERD can be treated medically and when there is poor response, an anti-reflux surgery is a therapeutic alternative. There are several case report series which document a recurrence rate of up to 21% [6] and dysphagia between 31–71% after the Nissen fundoplication [[7], [8], [9], [10]]. In the scleroderma context, a surgical approach is controversial given the ineffective esophageal motility ruling out a total Nissen fundoplication, some opting out for a partial Nissen fundoplication with recurrence rates of up to 19% [1]. For this reason, the surgical alternatives in GERD are limited. Other options include a total duodenal derivation [11] or an esophagectomy [12], both high morbidity approaches. Hence the Nissen fundoplication remains the standard, albeit controversial, surgical option in scleroderma patients.

In this case report, the advanced gastrointestinal disease secondary to scleroderma limited the standard surgical approach given the high recurrence rate. In this situation, an open gastrectomy with roux-en-Y anastomosis was decided as the best approach to solve the GERD and abide by the ISHLT standards for a lung transplant given the high risk of recurrence as well as potential complications given the comorbidities associated. The University of Pittsburgh first published the comparison between the laparoscopic Nissen fundoplication, the open gastrectomy with roux-en-Y anastomosis, and the esophagectomy in GERD patients secondary to scleroderma. They concluded that the gastrectomy approach had a lower rate of recurrence and lower reported dysphagia in the postoperative state compared to the other two alternatives [13,14].

## 4 Conclusions

**Fig. 6:**
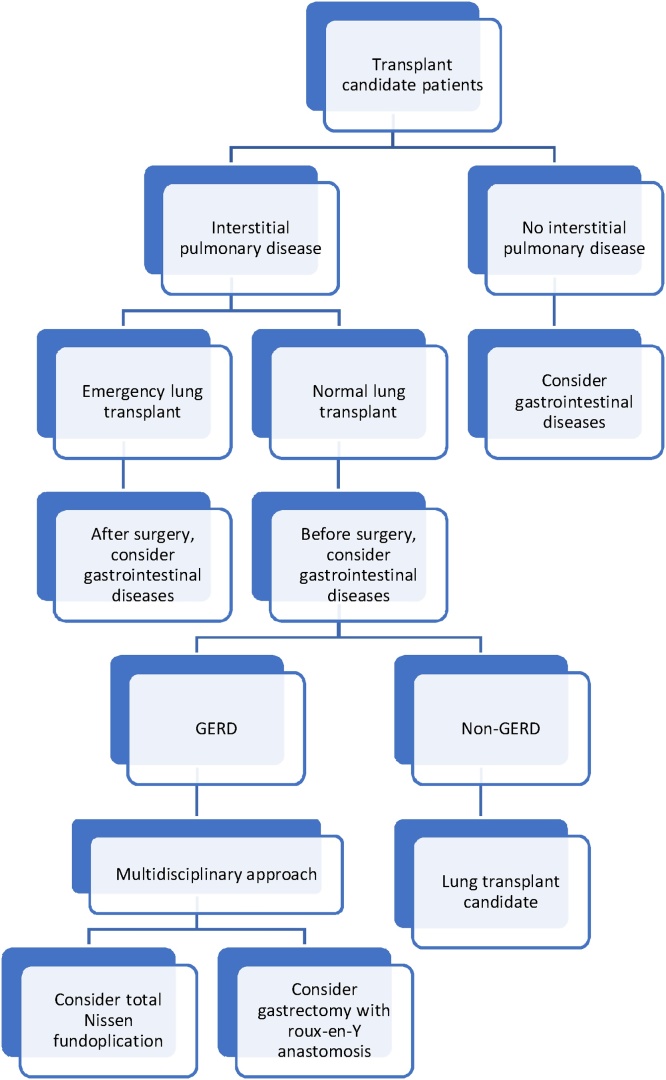
A propose protocol for evaluating gastrointestinal disease in lung transplant patients.

In summary, we propose an open gastrectomy with roux-en-Y anastomosis as a surgical alternative to the Nissen fundoplication in patients with advanced connective tissue disease. In Fig. 6, we also propose a new protocol for evaluating gastrointestinal disease in patients with a severe lung disease requiring lung transplant. The decision to provide a surgical intervention must be individualized, considering the expertise of the surgeons and transplant teams. An interdisciplinary treatment is very important, including a lung transplant and gastrointestinal team to ensure a successful intervention for these patients.

## Declaration of Competing Interest

The authors declare they have no conflicts of interest.

## Funding

This research did not receive any specific grant from funding agencies in the public, commercial, or not-for-profit sectors.

## Ethical approval

The Ethical and Research Committee of the Fundación Cardioinfantil – IC and the General Surgery Research Group at the Fundación Cardioinfantil – IC.

## Consent

Written consent was obtained from the patient for publication of this report. Any details identifying the individuals to the clinical history and images associated were eliminated as to remain anonymous.

## Author contribution

Perez Rivera CJ, González-Orozco A, Ocampo MA and Caicedo I designed the report, analyzed the data, and wrote the paper. Kadamani AA and Mosquera MS collected patient’s data and were the perioperative attending physicians.

## Registration of research studies

N/A.

## Guarantor

Perez Rivera Carlos Jose.

## Availability of data and material

The complete upper gastrointestinal endoscopes are available upon request by contacting the corresponding author.

## Provenance and peer review

Not commissioned, externally peer-reviewed.
